# Supplementary material for: Establishment and characterization of a cell line and patient-derived xenograft (PDX) from peritoneal metastasis of low-grade serous ovarian carcinoma
Source: Sci Rep. 2020 Apr 21;10:6688. doi: 10.1038/s41598-020-63738-6 (PMC7174384; doi:10.1038/s41598-020-63738-6)
Supplement: Supplementary file 1 — Supplementary Information. [file 41598_2020_63738_MOESM1_ESM.pdf]

Establishment and characterization of a cell line and patient-derived xenograft (PDX) from peritoneal metastasis of low-grade serous ovarian carcinoma

Authors:
Elien De Thaye[1,2], Koen Van de Vijver[2,3], Joni Van der Meulen[2,4], Joachim Taminau[2,5], Glenn Wagemans[2,6], Hannelore Denys[2,7], Jo Van Dorpe[2,3], Geert Berx[2,5], Wim Ceelen[2,8], Jan Van Bocxlaer[1], Olivier De Wever*[2,6]

Affiliation of authors:

(1) Laboratory of Medical Biochemistry and Clinical Analysis, Ghent University, Ghent, Belgium
(2) Cancer Research Institute Ghent (CRIG), Ghent, Belgium
(3) Department of Pathology, Ghent University Hospital, Ghent, Belgium
(4) Molecular Diagnostics Ghent University Hospital, Ghent, Belgium
(5) Molecular and Cellular Oncology lab, Ghent University, Ghent, Belgium
(6) Laboratory of Experimental Cancer Research, Ghent University, Ghent, Belgium
(7) Department of Medical Oncology, Ghent University Hospital, Ghent, Belgium
(8) Department of GI Surgery, Ghent University Hospital, Ghent, Belgium

Correspondence to: Olivier De Wever, Laboratory of Experimental Cancer Research, Department of Radiation Oncology and Experimental Cancer Research, Faculty of Medicine and Health Sciences, Ghent University, Corneel Heymanslaan 10, B-9000 Ghent, Belgium. Tel.: +32-9-3323073. Fax.: +32-9-3324991. E-mail: olivier.dewever@ugent.be

Supplementary information

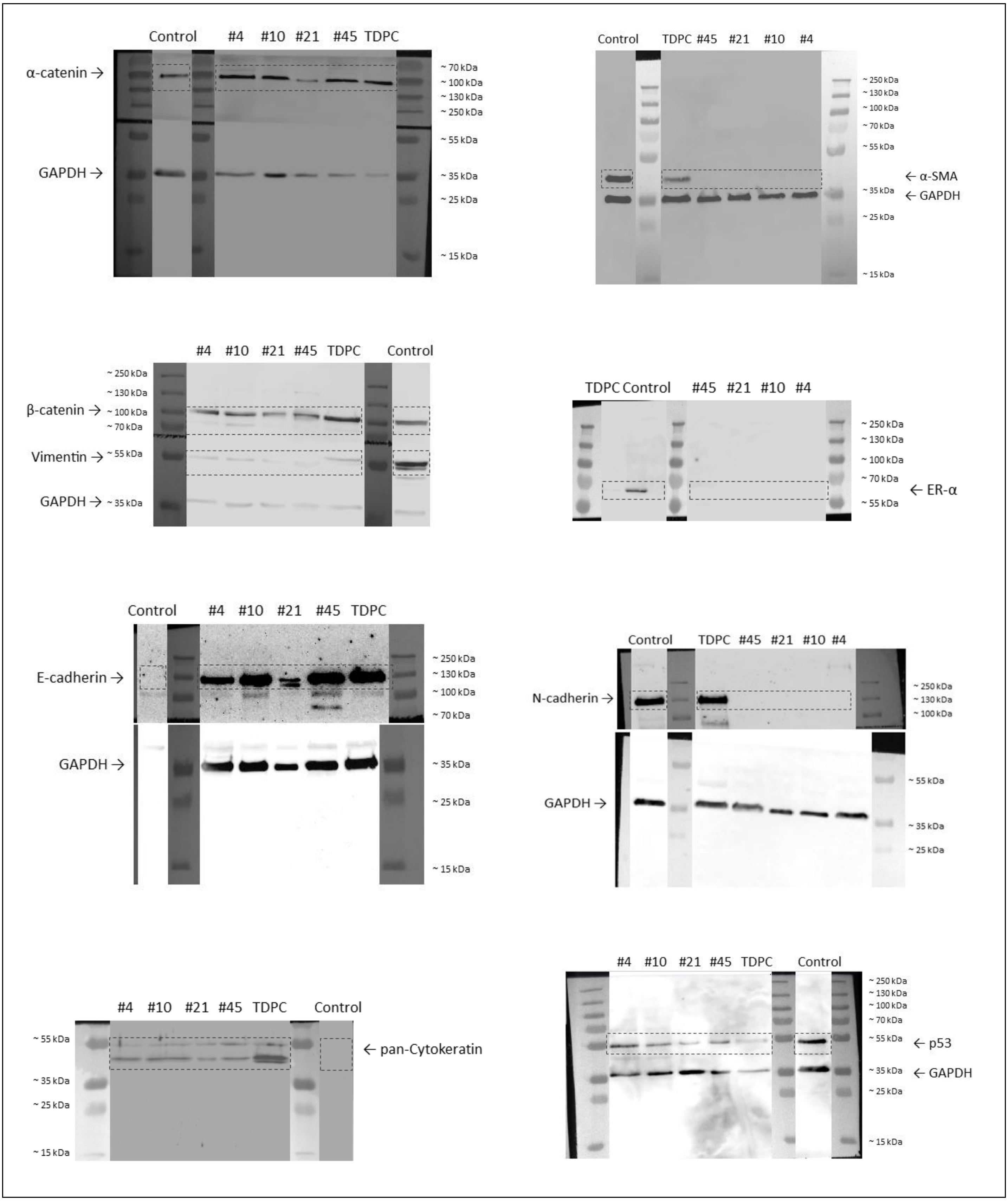

Supplementary Figure S1. Original western blot panels with gel markers for Figure 2B. Western blot images are overlaid onto image containing molecular weight markers. Black dashed lines identifies cropped region shown in respective figure (TDPC = tumor-derived primary cells).

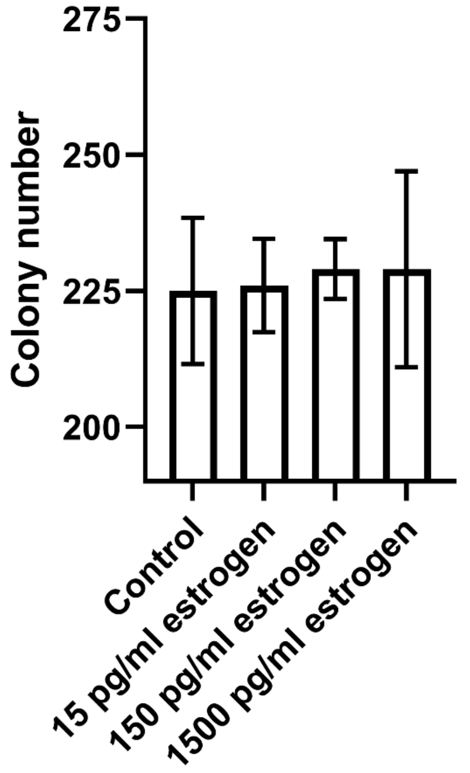

Supplementary Figure S2. No significant differences in PM-LGSOC-01 colony formation were found between the different estrogen conditions and the control condition tested. Data represent three replicates per condition (mean ± SEM).

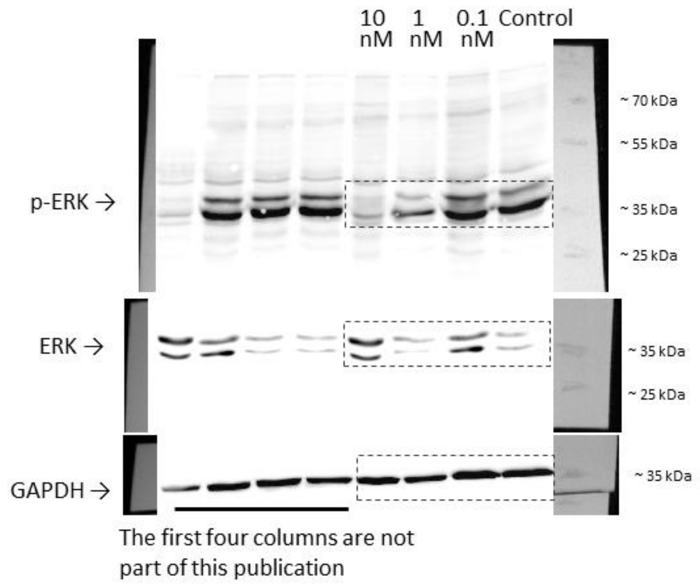

Supplementary Figure S3. Original western blot panels with gel markers for Figure 3B. Western blot images are overlaid onto image containing molecular weight markers. Black dashed lines identifies cropped region shown in respective figure (Concentrations in nM correspond to the trametinib treatment).

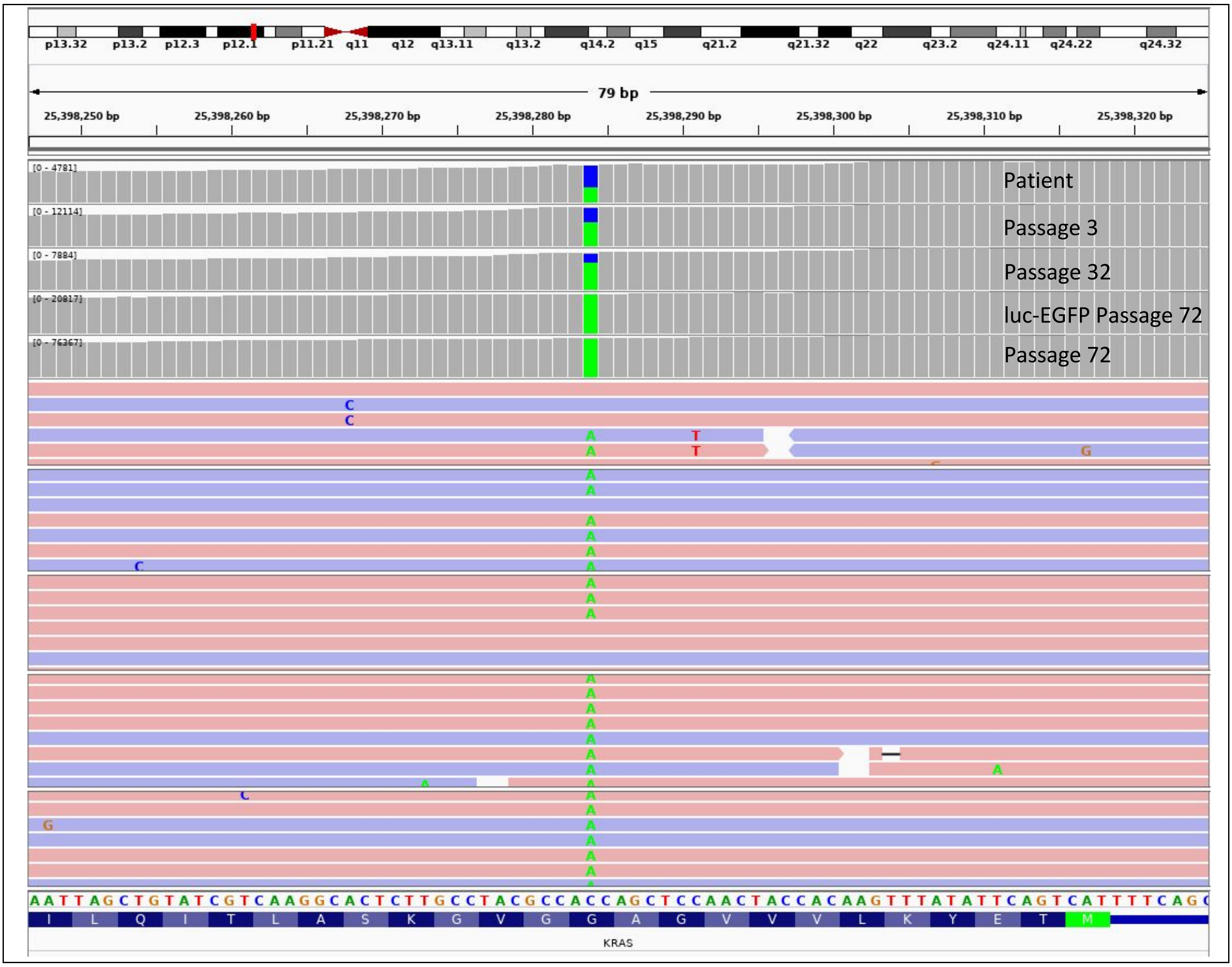

Supplementary Figure S4. *KRAS* c.35G>T (p.(Gly12Val)) mutation analysis at patient material, different in vitro passages of the PM-LGSOC-01 cell line (3, 32 and 72) and for the luc-EGFP positive PM-LGSOC-01 cells. Complete figure for Figure 3A.
